# Predictors of participation of adolescents with cerebral palsy: A European multi-centre longitudinal study

**DOI:** 10.1016/j.ridd.2014.10.043

**Published:** 2015-01

**Authors:** Van Mô Dang, Allan Colver, Heather O. Dickinson, Marco Marcelli, Susan I. Michelsen, Jackie Parkes, Kathryn Parkinson, Marion Rapp, Catherine Arnaud, Malin Nystrand, Jérôme Fauconnier

**Affiliations:** a UJF Grenoble 1/CNRS/CHU de Grenoble/TIMC-IMAG UMR 5525/Themas, Grenoble F-38041, France; b Institute of Health and Society, Newcastle University, Royal Victoria Infirmary, Newcastle upon Tyne NE1 4LP, UK; c AUSL Viterbo, Via Enrico Fermi 15, 01100 Viterbo, Italy; d National Institute of Public Health, Oster Farimagsgade 5, 1353 Copenhagen, Denmark; e School of Nursing & Midwifery, Queen's University Belfast, 21 Stranmillis Road, Belfast BT9 5AF, UK; f Klinik für Kinder und Jugendmedizin, Universität Lübeck, Ratzeburger Allee 160, 23538 Lübeck, Germany; g INSERM, UMR 1027, Paul Sabatier University, Toulouse, France; h Purpan, Clinical Epidemiology Unit, Toulouse, France; i Göteborg University, The Queen Silvia Children's Hospital, S-41685 Göteborg, Sweden

**Keywords:** Participation, Adolescence, Cerebral palsy, Longitudinal predictors

## Abstract

We investigated whether childhood factors that are amenable to intervention (parenting stress, child psychological problems and pain) predicted participation in daily activities and social roles of adolescents with cerebral palsy (CP). We randomly selected 1174 children aged 8–12 years from eight population-based registers of children with CP in six European countries; 743 (63%) agreed to participate. One further region recruited 75 children from multiple sources. These 818 children were visited at home at age 8–12 years, 594 (73%) agreed to follow-up at age 13–17 years.

We used the following measures: parent reported stress (Parenting Stress Index Short Form), their child's psychological difficulties (Strength and Difficulties Questionnaire) and frequency and severity of pain; either child or parent reported the child's participation (LIFE Habits questionnaire). We fitted a structural equation model to each of the participation domains, regressing participation in childhood and adolescence on parenting stress, child psychological problems and pain, and regressing adolescent factors on the corresponding childhood factors; models were adjusted for impairment, region, age and gender.

Pain in childhood predicted restricted adolescent participation in all domains except *Mealtimes* and *Communication* (standardized total indirect effects *β* −0.05 to −0.18, 0.01 < *p* < 0.05 to *p* < 0.001, depending on domain). Psychological problems in childhood predicted restricted adolescent participation in all domains of social roles, and in *Personal Care* and *Communication* (*β* −0.07 to −0.17, 0.001 < *p* < 0.01 to *p* < 0.001). Parenting stress in childhood predicted restricted adolescent participation in *Health Hygiene*, *Mobility* and *Relationships* (*β* −0.07 to −0.18, 0.001 < *p* < 0.01 to *p* < 0.001). These childhood factors predicted adolescent participation largely via their effects on childhood participation; though in some domains early psychological problems and parenting stress in childhood predicted adolescent participation largely through their persistence into adolescence.

We conclude that participation of adolescents with CP was predicted by early modifiable factors related to the child and family. Interventions for reduction of pain, psychological difficulties and parenting stress in childhood are justified not only for their intrinsic value, but also for probable benefits to childhood and adolescent participation.

## Highlights

• Pain in childhood predicted restricted adolescent participation in 10/11 domains.
• Psychological problems in childhood predicted restricted adolescent participation in all social roles, and in *Personal Care* and *Communication*.
• Parenting stress in childhood predicted restricted adolescent participation in *Health Hygiene*, *Mobility* and *Relationships*.
• These childhood factors predicted adolescent participation largely via their effects on childhood participation.

## 1 Introduction

Children with cerebral palsy (CP) experience restricted participation in life situations ranging from leisure pursuits to education and social roles (Beckung & Hagberg, 2002). Most children with CP live to adulthood, where they remain at higher risk of social disadvantage than adults without CP in terms of independent living, employment and establishing a family (Michelsen, Uldall, Hansen, & Madsen, 2006). Adolescence may be particularly challenging for young people with physical impairments (King, Brown, & Smith, 2003a). Delayed puberty, the psychological consequences of perception of body image, and fewer opportunities to socialise out of school may make this period more difficult. Medical care may be jeopardised as responsibility transfers from parent to young person and from child to adult health services.

Adolescents with CP have restricted participation in daily activities and social roles which depends on the severity of their impairments (Donkervoort, Roebroeck, Wiegerink, van der Heijden-Maessen, & Stam, 2007). Participation of children and adolescents with CP is associated with the modifiable factors: pain (Fauconnier et al., 2009), psychological problems (Ramstad, Jahnsen, Skjeldal, & Diseth, 2012) and parenting stress (Majnemer et al., 2008). However, evidence is scarce about the modifiable factors in childhood which predict participation in adolescence. A study with a longitudinal design (Holmbeck, Franks Bruno, & Jandasek, 2006) can help to distinguish participation patterns determined by factors operating in adolescence from patterns determined by factors already operating in childhood.

The objective of this paper is to evaluate how participation of adolescents with CP is associated with modifiable childhood factors: pain, psychological problems, and parenting stress. We studied whether these associations were mediated by participation in childhood or by the level of the same predictors in adolescence.

## 2 Methods

### 2.1 Setting and participants

The present work is part of a larger project, SPARCLE, which studies the participation and quality of life of children and adolescents with CP in Europe. The overall design of the project, including sample size calculations, is described elsewhere (Colver and Dickinson, 2010, Colver, 2006) and is summarised below.

Children born between 31/07/1991 and 01/04/1997 were randomly sampled from population-based registers of children with CP in eight European regions (Table 1) that share a standardised definition of CP (Surveillance of Cerebral Palsy in Europe (SCPE), 2000). 743/1174 (63%) target families identified from registers joined the study. One further region, northwest Germany, ascertained 75 cases from multiple sources, using the same diagnostic criteria. The 818 children who entered the study were interviewed initially in 2004/2005, aged 8–12 years (SPARCLE1), and followed up in 2009/10, aged 13–17 years (SPARCLE2), when 594 (73%) remained in the study. Predictors of drop-out have been reported (Dickinson et al., 2006, Dickinson et al., 2012). Researchers from the nine regions visited families in their homes to administer questionnaires to parents and their children. The researchers had attended common training in order to maximise homogeneity of survey methodology across regions.

**Table 1:** Distribution of predictors of participation.

	Childhood *n* (%)
*(a) Impairment in childhood*	
Walking ability (GMFCS)	
I Child walks and climbs stairs	176 (30)
II Child walks inside	132 (22)
III Child walks with limitations	102 (17)
IV Moving about is limited	85 (14)
V Moving about is severely limited	99 (17)
Missing	0 (0)
Two-handed fine motor function (BFMF)	
I Without limitation	201 (34)
II Both hands limited in fine skills	162 (27)
III Child needs help with tasks	95 (16)
IV Child needs help and adapted equipment	71 (12)
V Child needs total human assistance	65 (11)
Missing	0 (0)
Seizures (in previous year)	
No seizures and not on medication	427 (72)
No seizures and on medication	55 (9)
Seizures less than once a month	48 (8)
Seizures more than once a month and less than once a week	32 (5)
Seizures more than once a week	32 (5)
Missing	0 (0)
Feeding	
Feeds by mouth with no problems	429 (72)
Feeds by mouth but with difficulty	131 (22)
Partial or complete feeding by tube	34 (6)
Missing	0 (0)
Communication	
Normal communication	341 (57)
Problem but communicates with speech	102 (17)
Uses alternative formal methods to communicate	73 (12)
No formal communication	78 (13)
Missing	0 (0)
Intellectual impairment (IQ)	
>70	289 (49)
50–70	138 (23)
<50	162 (28)
Missing	5 (1)

Predictor	Childhood *n* (%)	Adolescence *n* (%)
*(b) Pain, psychological problems, parenting stress*		
Pain frequency		
None of the time	174 (30)	158 (27)
Once or twice, or a few times	308 (53)	276 (47)
Fairly often, very often, or almost every day or every day	104 (18)	152 (26)
Missing	8 (1)	8 (1)
Pain severity		
None	175 (30)	158 (27)
Very mild or mild	263 (45)	211 (36)
Moderate, severe or very severe	148 (25)	217 (37)
Missing	8 (1)	8 (1)
Total difficulties score of SDQ		
Normal 0–13	350 (60)	357 (61)
Borderline 14–16	107 (18)	106 (18)
Abnormal 17–40	130 (22)	125 (21)
Missing	7 (1)	6 (1)
Total stress score of PSI-SF		
Normal (<85)	62 (10)	49 (8)
Borderline (85–90)	335 (56)	332 (56)
>90	180 (31)	200 (34)
Missing	17 (3)	13 (2)

### 2.2 Measures

We evaluated participation using the questionnaire of Life Habits (LIFE-H) (Noreau et al., 2004) which is based on a social model of disability similar to the theoretical framework of the World Health Organisation's International Classification of Functioning (World Health Organisation, 2007) and has been validated in children with disabilities (Noreau et al., 2007). Wherever possible the adolescent completed the questionnaire; otherwise a parent completed it. It consists of 62 items divided into six domains of daily life activities (*Mealtimes*, *Health hygiene*, *Personal care*, *Communication*, *Home life*, and *Mobility*) and five domains of social roles (*Responsibilities*, *Relationships*, *Community life*, *School*, and *Recreation*). It includes fifteen “non-discretionary” activities, such as transferring into or out of bed, which are essential for daily living; and forty-seven further “discretionary” activities, such as exercise to optimise health, which may or may not be achieved.

We recorded the discretionary items using three levels: not achieved because too difficult; achieved with difficulty; achieved without difficulty. A discretionary item could also be considered non-applicable if it was irrelevant, for example if the child or adolescent had no interest in that activity; we treated non-applicable items as missing responses. We recorded non-discretionary items in childhood using two levels (achieved with difficulty, achieved without difficulty) but in adolescence we used three levels (achieved with much difficulty, achieved with some difficulty, achieved without difficulty). The Life-H asks, for each item, how much assistance the young person requires, but we ignored this information because we wanted to assess participation without incorporating the influence of environmental factors (Fauconnier et al., 2009).

In order to assess pain, we asked parents about the frequency and severity of their child's pain over the previous week; we recorded responses on six levels, but grouped them into three categories for analysis. We captured the psychological problems of the child or adolescent using the Total Difficulties Score of the parent-reported Strength and Difficulties Questionnaire (SDQ) (Goodman, 1997). We captured parenting stress using the Total Stress Score of the Parenting Stress Index Short Form (PSI-SF) (Abidin, 1995). Parents provided information about their child's impairments (walking ability as captured by the gross motor function classification system (GMFCS) (Palisano et al., 1997), fine motor function (Beckung & Hagberg, 2002), seizures, feeding, communication, intellectual ability (White-Koning et al., 2005)), family structure and parents’ educational qualifications.

### 2.3 Statistical methods

Full details of the statistical methods are reported in Appendix Statistics and summarised below.

For each participation domain, we translated hypotheses about the variables that might influence participation into one structural equation model which comprised a ‘measurement part’ that defined the latent constructs that underlie sets of observed variables; and a ‘structural part’ that hypothesised the links between these constructs.

**Fig. 1:**
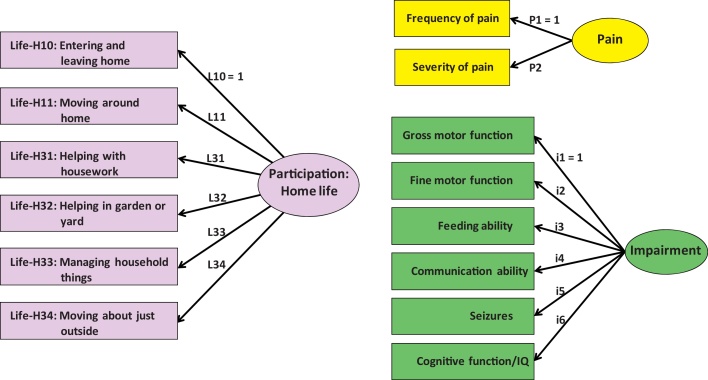
Measurement models for domains of participation (illustrated by *Home Life*), pain and impairment.

The measurement part (illustrated in Fig. 1 for one domain, *Home life*) considered participation in each domain to be an unobserved or latent variable, manifested by responses to the items in the LIFE-H questionnaire. We likewise modelled impairment and pain using latent variables, manifested respectively by the levels of the six individual impairments as recorded in childhood and by the frequency and severity of pain over the previous week (see Fig. 1).

**Fig. 2:**
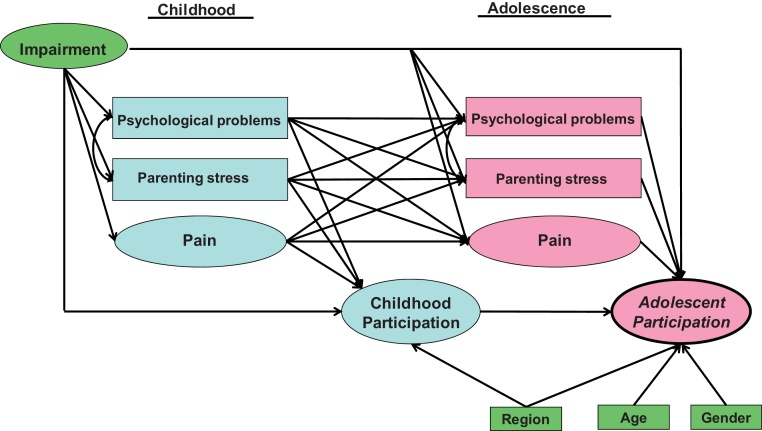
Structural model applied to each domain of participation with postulated relationships between modifiable factors and participation. Variables within ellipses are latent, defined in the measurement model of Fig. 1; variables within rectangles are observed. Straight lines indicate direct effects; curved lines indicate correlations. Adjusting variables are in green, childhood variables in blue and adolescent variables in pink.

The structural part specified the hypothesised links between the variables, both latent and observed (see Fig. 2). For each participation domain, we hypothesised that participation in both childhood and adolescence may be directly affected by the concurrent factors: pain (Doralp & Bartlett, 2010), psychological problems (Ramstad et al., 2012), and parenting stress (Blum, Resnick, Nelson, & St Germaine, 1991). Additionally, we hypothesised that each factor in childhood could indirectly affect participation in adolescence via its influence on four mediating variables: participation in childhood, and the three factors in adolescence. We adjusted for impairment, region, gender, and age. Psychological problems, parenting stress, region, gender, and age were treated as observed variables; region and gender were categorical while the others were continuous.

Our analysis followed the steps outlined by Kline (2011) for a structural equation model. We assessed model goodness of fit by the root mean square error of approximation (RMSEA), which indicates a good fit when RMSEA <0.05. We identified the childhood factors that were significantly related to each domain of childhood participation through preliminary analysis that was restricted to childhood variables. We then developed the full model, retaining only these significant childhood factors and similarly identifying adolescent factors that were significantly related to adolescent participation.

We report the estimated indirect effects (*β*) of childhood factors (pain, psychological problems, parenting stress) on adolescent participation. The *total indirect effects* were the sum of the *partial indirect effects* via all four possible pathways (see Figures and Appendix Statistics). These indirect effects were standardised in order to compare the contributions of different predictors to participation; a standardised effect of *β* of a predictor on a specific outcome means that an increase of one standard deviation in the predictor is associated with a change of *β* standard deviations in the outcome. As it was of interest to compare the effect of modifiable childhood factors with the effect of impairment, we also noted the standardised direct, indirect and total effects of impairment on adolescent participation. We determined statistical significance (*p*-values) from the estimated standard errors of the unstandardised effects. Finally we undertook sensitivity analyses around drop-out, including all 818 SPARCLE1 participants and imputing missing data (van Buuren, 2007).

We analysed the data using Mplus software, version 6.12 (Muthén & Muthén, 1998).

### 2.4 Ethics

In each country, we obtained ethical approval or a statement that only registration was required, as appropriate. We obtained signed consent from all parents and from young people who could give meaningful consent.

## 3 Results

The average age of the children was 10.4 years at the first visit and 15.1 years at the second visit; 249 (42%) were girls. The average time between visits was 4.7 years (3.6–5.8 years).

Table 1 shows the distribution of putative predictors of participation. The rate of missing data was low: not more than 3% for any variable. Parents reported that approximately two thirds of their children experienced at least some pain during the previous week. The Total Difficulties Score of SDQ was abnormal (>16) in 21% of adolescents, a proportion twice that in the general population (Goodman, 1997). The Total Stress Score of PSI-SF was abnormal (>90) for 34% of their parents, twice the proportion in the general population (Abidin, 1995).

Table 2 shows the distribution of participation items for each age group. Non-response remained less than 3% except for the item of extra classes in childhood. Among adolescents, discretionary items were considered non-applicable by between 0% (getting a good sleep) and 52% (religious activities). Both items of the *Community life* domain were considered non-applicable by 26%, with marked differences between regions, so we excluded that domain from analysis as in our prior study (Fauconnier et al., 2009). The proportion of adolescents achieving an item without difficulty varied widely, from 31% (moving on slippery or uneven surfaces) to 93% (maintaining a loving relationship with one's parents).

**Table 2:** Distribution of participation items.




^a^Not applicable: number of respondents who considered the (discretionary) activity to be non-applicable.

*Non-discretionary activity.

In preliminary investigations, we fitted the measurement model for each domain and generated participation scores for each adolescent. These varied significantly between regions for all domains except *Relationships*; excluding this domain, the variation between regions was 3–11% of the total variation in participation, depending on domain; in contrast, the variation between levels of GMFCS was 22–60% of the total variation.

### 3.1 Predictors of adolescent participation

**Fig. 3:**
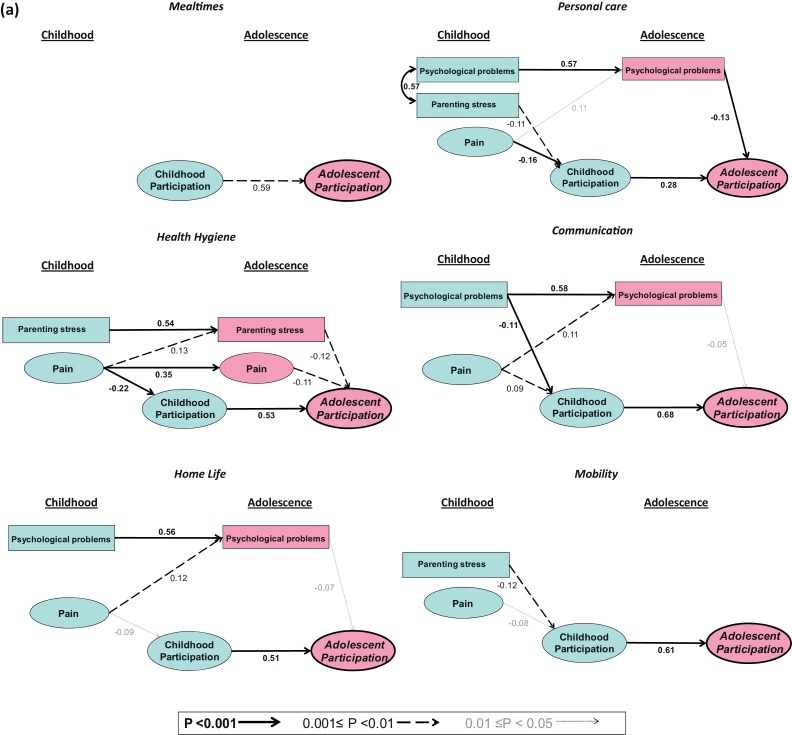

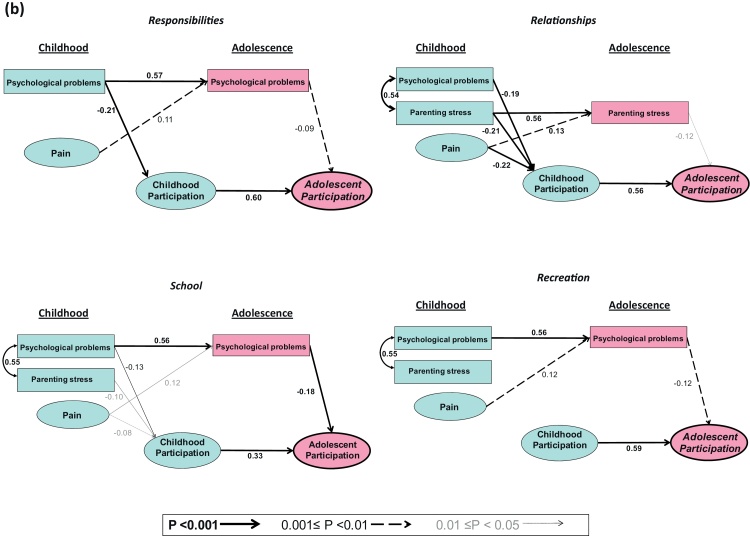
Final structural models for domains of daily life activities (3a) and Social roles (3b). The models were additionally adjusted for gender, age, region and impairment (see Appendix Statistics). The direct and indirect effects of impairment are reported in Table 3. Variables within ellipses are latent, defined in the measurement model of Fig. 1; variables within rectangles are observed. Straight lines indicate direct effects; curved lines indicate correlations. Numerical values are standardised regression coefficients for each direct path.

Table 3 and Fig. 3 summarise the estimated effects of early predictors on adolescent participation. The goodness of fit of the models was good (0.037 ≤ RMSEA ≤ 0.048). The models explained between 61% and 90% of the variance of participation in adolescence, except in the *Relationships* domain where the model explained only 41%.

**Table 3:** Standardised effects of childhood predictors and impairment on adolescent participation.^a^

	Mealtimes	Health hygiene	Personal care	Communication	Home life	Mobility
*(a) Daily life activities*						
bRMSEA	0.039	0.046	0.038	0.048	0.038	0.041
c*R*^2^	0.90	0.82	0.67	0.88	0.82	0.77
**Indirect effects of childhood predictors**						
Childhood pain – total		−0.18***	−0.06**	0.06**	−0.05**	−0.05*
- Via childhood participation		−0.12***	−0.05**	0.06**	−0.05*	−0.05*
- Via adolescent pain		−0.04**				
- Via adolescent psychological problems			−0.01*		−0.01.	
- Via adolescent parenting stress		−0.02*				
Childhood psychological problem – total			−0.07***	−0.11***	−0.04*	
- Via childhood participation				−0.07***		
- Via adolescent pain						
- Via adolescent psychological problems			−0.07***	−0.03*	−0.04*	
- Via adolescent parenting stress						
Childhood parenting stress – total		−0.11***	−0.04**			−0.07**
- Via childhood participation		−0.04.	−0.03*			−0.07**
- Via adolescent pain						
- Via adolescent psychological problems						
- Via adolescent parenting stress		−0.07**				
**Direct and indirect effects of impairment**						
Total effect	−0.88***	−0.79***	−0.69***	−0.87***	−0.81***	−0.75***
- Direct effect	−0.39*	−0.29**	−0.51***	−0.29***	−0.41***	−0.42***
- Indirect effect via childhood participation^d^	−0.48**	−0.37***	−0.15***	−0.58***	−0.37***	−0.29***

	Responsibilities	Relationships	School	Recreation
*(b) Social roles*				
RMSEA	0.043	0.043	0.045	0.037
*R*^2^	0.88	0.41	0.61	0.76
**Indirect effects of childhood predictors**				
Childhood pain – total	−0.01*	−0.14***	−0.05**	−0.05*
- Via childhood participation		−0.12***	−0.03.	−0.04.
- Via adolescent pain				
- Via adolescent psychological problems	−0.01*		−0.02*	−0.01*
- Via adolescent parenting stress		−0.02.		
Childhood psychological problems - total	−0.17***	−0.11**	−0.14***	−0.11***
- Via childhood participation	−0.12***	−0.11**	−0.04**	−0.04.
- Via adolescent pain				
- Via adolescent psychological problems	−0.05**		−0.10***	−0.07**
- Via adolescent parenting stress				
Childhood parenting stress – total		−0.18***	−0.05*	−0.05*
- Via childhood participation		−0.11**	−0.03*	−0.04.
- Via adolescent pain				
- Via adolescent psychological problems			−0.01.	−0.01.
- Via adolescent parenting stress		−0.07*		
**Direct and indirect effects of impairment**				
Total effect	−0.84***	−0.37***	−0.63***	−0.72***
- Direct effect	−0.35***	−0.03.	−0.42***	−0.31***
- Indirect effect via childhood participation^d^	−0.47***	−0.21***	−0.17***	−0.36***

a The model controlled for region, gender and age. Statistical significance:

* 0.01 < *p* < 0.05.

** 0.001 < *p* < 0.01.

*** *p* < 0.001.

. *p* > 0.5

b RMSEA = goodness of fit index of the estimated model.

c *R*^2^ = proportion of latent adolescent participation variance explained by the model.

d Indirect effects of impairment on adolescent participation via pathways that involved child and adolescent pain, psychological problems and parenting stress were generally negligible.

As expected, impairment predicted a significant restriction of adolescent participation in all domains, with standardised total effects ranging from *β* = −0.37 (*p* < 0.001) for *Relationships* to *β* = −0.88 (*p* < 0.001) for *Mealtimes.*

Pain in childhood predicted a significant restriction of adolescent participation in all domains except *Mealtimes*; the size of its standardised indirect effect was most marked in *Health hygiene* (*β* = −0.18, *p* < 0.001) and *Relationships* (*β* = −0.14, *p* < 0.001) (indicating that an increase of one standard deviation in childhood pain was associated with decreases of 0.18 and 0.14 standard deviations respectively in these domains of participation). Psychological problems in childhood predicted a significant restriction in adolescent participation in all domains of social roles, effects ranging from *β* = −0.11 in *Relationships* (0.001 < *p* < 0.01) to *β* = −0.17 in *Responsibilities* (*p* < 0.001), and in the daily life activities of *Personal care* and *Communication* (*β* = −0.07 and −0.11 respectively, *p* < 0.001). Parenting stress in childhood predicted restricted adolescent participation in *Health hygiene* (*β* = −0.11, *p* < 0.001), *Mobility* (*β* = −0.07, 0.001 < *p* < 0.01) and *Relationships* (*β* = −0.18, *p* < 0.001). Adolescent participation in *Mealtimes* was not significantly associated with any of the childhood predictors.

### 3.2 Pathways to participation

Direct and indirect effects of impairment were of similar magnitude in most domains, the indirect effects being mediated largely by childhood participation.

The associations between modifiable childhood predictors and adolescent participation were largely mediated by childhood participation, which was a strong predictor of adolescent participation in most domains: a change of one standard deviation in childhood participation predicted a change of between 0.28 and 0.68 standard deviations in adolescent participation, depending on domain (see Fig. 3). The influence of childhood pain on adolescent participation was essentially mediated via its direct effect on childhood participation (see Table 3 and Fig. 3); the partial indirect effects of childhood pain via adolescent factors were generally small or negligible (partial *β* ≤ 0.04). Psychological problems in childhood predicted adolescent participation in *Communication*, *Responsibilities* and *Relationships* mainly via child participation (partial *β* = −0.07, −0.12 and −0.11 respectively) but they predicted adolescent participation in the domains of *Personal care*, *School* and *Recreation* mainly via psychological problems in adolescence. Parenting stress in childhood was also significantly related to adolescent participation in *Mobility* and *Relationships* via childhood participation (partial *β* = −0.07 and −0.11 respectively) but via parenting stress during adolescence in the domains of *Health hygiene* and *Relationships*.

Sensitivity analysis, which imputed missing data for those who dropped out between childhood and adolescence, yielded similar results (data not shown).

## 4 Discussion

Childhood participation was the main predictor of adolescent participation (a change of one standard deviation in childhood participation predicted a change of between 0.28 and 0.68 standard deviations in adolescent participation, depending on domain). Three factors in childhood (pain, psychological problems and parenting stress) predicted, in varying degrees, restricted participation at adolescence in all domains except *Mealtimes*. However, these effects were small: a change of one standard deviation in any of the three childhood factors predicted a change of at most 0.18 standard deviations in adolescent participation. Furthermore, these three childhood factors predicted adolescent participation largely via their effects on childhood participation, although in some domains early psychological problems and parenting stress in childhood affected adolescent participation through their persistence into adolescence. Effects of impairment were much larger than the effects of these childhood factors: a difference of one standard deviation in impairment was associated with a difference of more than 0.6 standard deviations in adolescent participation in all domains except *Relationships*, the main pathway again being via childhood participation.

### 4.1 Strengths and limitations

Because sampling of the children was multinational and from population registers of children with CP, conclusions may be generalised to the population of adolescents with CP living in Europe.

As in any regression, statistical associations do not prove causation. Unmeasured, shared causes could explain part of the associations; for instance parenting style may influence both parenting stress and participation. Although we based our hypothesised directions of effects on prior research (Dang, 2012), alternative directions of effects should be considered (King et al., 2003b). For instance, increased participation may improve the psychological health of the child (Dahan-Oliel, Shikako-Thomas, & Majnemer, 2012).

Non-response by families targeted for recruitment to SPARCLE1 was 37% (Dickinson et al., 2006), and drop-out between SPARCLE1 and SPARCLE2 was 27% (Dickinson et al., 2012). The parents who dropped out between SPARCLE1 and SPARCLE2 had a higher level of stress when their children were aged 8–12 than those retained in the study. Although such differential non-response is likely to result in biased estimates of population means, it may be less important in the present study which estimates associations (Korn & Graubard, 1999). Nevertheless, we tried to minimise the effects of differential non-response and drop-out in two ways. Firstly, we adjusted for region and walking ability, which were predictors of non-response (Dickinson et al., 2006, Korn and Graubard, 1999). Secondly, we performed a sensitivity analysis which included all children who participated in SPARCLE1, imputing missing data; this yielded similar results to the primary analysis.

We considered that the young person knew most about their participation; but if a young person could not self-report due to intellectual impairment we relied on parent-report. Parent-reported pain may differ from self-reported pain (Parkinson, Gibson, Dickinson, & Colver, 2010), but we chose it in order to have a common metric across the sample. Prior research has tended to examine the impacts of specific impairments on activities (Beckung and Hagberg, 2002, Fauconnier et al., 2009), but in our study we controlled for impairment using a single latent variable because children with CP are affected by several correlated functional limitations reflecting the global severity of the cerebral disturbance. The model did not explicitly take account of environmental influences such as the availability of specialised schools or the accessibility of transportation; however, by controlling for region, we took account of the regional variation in environments. As in any structural equation approach, other models may fit the data equally well (Kline, 2011).

The prevalence of the predictors of participation in our sample was comparable to that in other studies; for example, pain was present in 50–70% of children and adolescents with CP (Doralp and Bartlett, 2010, Engel et al., 2005, Hadden and von Baeyer, 2002), psychological problems in 39% to 54% of children with CP (Brossard-Racine et al., 2012, Goodman and Graham, 1996), and symptoms of stress in about 30% of mothers of children with CP (Manuel et al., 2003, Sawyer et al., 2011).

### 4.2 Comparison with other studies

We could not find other longitudinal studies of early predictors of participation of adolescents with disabilities. However, King et al. (2009) examined predictors of change in intensity of participation in leisure and recreational activities over a three year period of children with physical disabilities aged 6–15. They found that participation intensity declined over the years in recreational, active physical and social activities but not in skill-based and self-improvement activities. Factors associated with these changes varied with type of activity and the child's age and sex. Their conclusions emphasised individual variability and proposed that interventions should be tailored to the individual child. It is difficult to compare their study with ours, because they measured the intensity (frequency) of participation in leisure activities and therefore could not address, as we did, difficulty in participation in essential daily activities such as feeding and toileting.

Our findings are consistent with findings from cross-sectional studies that pain limits daily activities in children (Tervo, Symons, Stout, & Novacheck, 2006) and adolescents with CP (Doralp & Bartlett, 2010). Pain reduces children's school attendance (Houlihan, O’Donnell, Conaway, & Stevenson, 2004) and predicts altered school functioning via fatigue (Berrin et al., 2007). Psychological problems in childhood may contribute to friendlessness and restricted participation in social roles (Doll, 1996), and be associated with decreased participation in children with CP (Ramstad et al., 2012). Parenting stress is associated with more coercive parent–child interactions (Plant & Sanders, 2007), and this could restrict the freedom of the child or adolescent to experiment with activities.

Other studies of children and adolescents with disabilities have highlighted the range and complex inter-relationships of child, family and community factors that predict participation (Colver et al., 2012, King et al., 2003b, King et al., 2006, King et al., 2009, Orlin et al., 2010, Yeung and Towers, 2014). In particular, King et al. (2006) used structural equation modelling to undertake a cross sectional analysis of children aged 6–14 years with physical disabilities, including CP, to examine child, family and environmental influences on leisure and recreational participation. Family participation in social and recreational activities influenced the child's participation, but the standardised *β* coefficient was only 0.18. Child preferences had a stronger *β* coefficient of 0.28 but this may reflect not only the personality and interests of the child but also environmental factors; for example, a child that has experienced unfriendliness in leisure settings will prefer not to attend such settings (Colver, 2010). King et al. (2006) found only a small indirect effect of an unsupportive environment; however this small effect may be partly because they used CHIEF to measure the physical, social and attitudinal environment; CHIEF is a subjective measure of the frequency and extent of perceived environmental barriers on participation rather than a direct measure of the environment; it may therefore reflect differing expectations of participation rather than actual environmental barriers.

In our study of the cohort aged 8–12, we found that their physical, social, and attitudinal environment influenced their participation in everyday activities and social roles (Colver et al., 2011, Fauconnier et al., 2009, Michelsen et al., 2009); the variation in adolescent participation between regions suggests environment is also an important influence on adolescent participation in several domains.

### 4.3 Implications for clinical practice

Important modifiable predictors of participation of adolescents with CP are pain, psychological problems and parenting stress in childhood, which are highly prevalent in families with a child with CP. The consistency of the associations we have found in this longitudinal study and their correspondence to clinical expectation suggest that these factors have a causal role. These childhood factors influence adolescent participation largely via their influence on childhood participation, which strongly predicts adolescent participation, and to a lesser extent via their influence on the corresponding adolescent factors.

These findings highlight the importance of improving childhood participation in order to improve adolescent participation. Clinicians will want to intervene early to reduce pain, parenting stress and psychological problems, not only because these factors are intrinsically distressing but because of their likely effect on child participation. Pain management and the psychological aspect of pain in children with CP may be addressed by working on coping strategies (Jensen, Engel, & Schwartz, 2006). Psychological problems experienced by the child can be addressed (Beale, 2006) and interventions which target the family as a whole may improve the emotional and psychological symptoms of children with chronic conditions (Barlow & Ellard, 2004). Parenting stress can also be addressed directly (Barakat and Linney, 1992, Frey et al., 1989, Hinojosa and Anderson, 1991). Ideally, multidisciplinary care should start early in childhood and continue into adolescence.

### 4.4 Implications for research

The most reliable way to assess whether the identified associations represent causal mechanisms would be to undertake randomised controlled trials of interventions to reduce child pain, child psychological problems and parenting stress, with long-term follow-up and measurement of participation as a secondary outcome. Trials of interventions aiming directly to improve participation are also needed as. Additionally, further follow-up of the same cohort would enable assessment of the long-term effects of childhood and adolescent precursors on adult participation. Ideally, future observational studies should have a sufficiently large sample size (e.g. over 1000 participants) to allow the use of person-centred analytic methods that identify different patterns of participation and their predictors (Bartko and Eccles, 2003, Shanahan and Flaherty, 2001).

## Role of the funding source

SPARCLE 1 (visits in childhood) was funded by the European Union Research Framework 5 Program – Grant number QLG5-CT-2002-00636, the German Ministry of Health GRR-58640-2/14 and the German Foundation for the Disabled Child.

SPARCLE 2 (visits in adolescence) was funded by: 10.13039/100004440Wellcome Trust WT 086315 A1A (UK & Ireland); Medical Faculty of the University of Lübeck E40-2009 and E26-2010 (Germany); CNSA, INSERM, MiRe – DREES, IRESP (France); Ludvig and Sara Elsass Foundation, The Spastics Society and Vanforefonden (Denmark); Cooperativa Sociale “Gli Anni in Tasca” and Fondazione Carivit, Viterbo (Italy); Goteborg University – Riksforbundet for Rorelsehindrade Barn och Ungdomar and the Folke Bernadotte Foundation (Sweden).

None of the above funders had any say in the design and conduct of the study; collection, management, analysis, and interpretation of the data; and preparation, review, or approval of the manuscript.

## Conflicts of interest

All authors declare that they have no conflicts of interest, including financial, personal or other relationships that could be perceived to influence this paper.

## Appendix Statistics

Measurement model: This is illustrated in Fig. 1. We used an ordered probit function to link the latent variables for participation, impairment and pain to their indicators (Muthen, 1984). For participation and pain, similar models were assumed in childhood and adolescence. Since latent variables have no scale, one of the loadings was arbitrarily constrained to equal 1 so that the model was identifiable.

Structural model: The hypothesised relationships between contextual factors and participation are illustrated in Fig. 2. Region, gender, and age were treated as observed variables; impairment was a latent variable. Finally, a residual correlation was modelled between concurrent psychological problems and parenting stress. We estimated the model parameters using the weighted least squares mean and variance adjusted algorithm (Muthén, Du Toit, & Spisic, 1997) with pairwise deletion of missing data; the estimator is consistent if missingness occurs at random depending only on observed covariates (Asparouhov and Muthén, 2010). We assumed that participation in a given domain could be independently affected by a subset of the three concurrent contextual factors, which was selected by applying two hierarchical ascendant regressions in each domain of participation, first on the sub-model of childhood participation and contextual factors, then on the full model. We compared hierarchical regression models using a chi-square difference test adjusted by the means and variances of parameters (Satorra & Bentler, 2001). Region, impairment, age, and gender were forced into the model irrespective of their estimated significance.

Finally we undertook sensitivity analyses to assess the possible effect of drop-out between SPARCLE1 and SPARCLE2. We generated five imputed datasets, using multiple imputation with chained equations (van Buuren, 2007) for all 818 young people who participated in SPARCLE1. Missing values of impairment, Life-H and pain responses, PSI and SDQ scores were imputed from values observed in SPARCLE1 for age, gender, PSI, SDQ and predictors of drop-out – region, walking ability, family structure and parental educational qualifications (Dickinson et al., 2006), using polytomous regression for categorical variables and predictive mean matching for interval scaled variables. We then analysed each imputed dataset using the methods described for the primary analysis. As it was not possible to combine these results for standardised effects or indirect effects, we noted the range of results (corresponding to Table 3) for each imputation.

Summary statistics: Our primary objective was to estimate the ‘total indirect effects’ *β* of childhood factors (pain, psychological problems, and parenting stress) on adolescent participation. These total indirect effects were the sum of the ‘partial indirect effects’ (partial *β*) via each possible pathway i.e. via both childhood participation and adolescent contextual factors. The partial indirect effect via each pathway was the product of the two regression coefficients on the direct paths (Kline, 2011). Estimated total and partial indirect effects are reported in Table 3; estimated regression coefficients are reported in Fig. 3.

Direct and indirect effects and regression coefficients were standardised in order to facilitate comparison of the contributions of different predictors.
